# Development and validation of a nomogram to predict survival outcome among epithelial ovarian cancer patients with site-distant metastases: a population-based study

**DOI:** 10.1186/s12885-021-07977-4

**Published:** 2021-05-25

**Authors:** Bo Wang, Shixuan Wang, Wu Ren

**Affiliations:** Department of Obstetrics and Gynecology, Tongji Hospital, Tongji Medical College, Huazhong University of Science and Technology, Wuhan, 430030 People’s Republic of China

**Keywords:** Epithelial ovarian cancer, Distant metastases, Prognosis, Nomogram score

## Abstract

**Background:**

Increasing evidence indicates that site-distant metastases are associated with survival outcomes in patients with epithelial ovarian cancer. This study aimed to investigate the prognostic values of site-distant metastases and clinical factors and develop a prognostic nomogram score individually predicting overall survival (OS, equivalent to all-cause mortality) and cancer specific survival (CSS, equivalent to cancer-specific mortality) in patients with epithelial ovarian cancer.

**Methods:**

We retrospectively collected data on patients with epithelial ovarian cancer from the Surveillance, Epidemiology, and End Results (SEER) database between 1975 and 2016. Multivariate Cox regression was performed to identify survival trajectories. A nomogram score was used to predict long-term survival probability. A comparison between the nomogram and the International Federation of Gynecology and Obstetrics (FIGO 2018) staging system was conducted using time-dependent receiver operating characteristic (tROC) curve.

**Results:**

A total of 131,050 patients were included, 18.2, 7.8 and 66.1% had localized, regional and distant metastases, respectively. Multivariate analysis identified several prognostic factors for OS including race, grade, histology, FIGO staging, surgery, bone metastasis, liver metastasis, lung metastasis, and lymphatic metastasis. Prognostic factors for CSS included grade, site, FIGO staging, surgery, bone metastasis, brain metastasis, lung metastasis, lymphatic metastasis, and insurance. Following bootstrap correction, the C-index of OS and CSS was 0.791 and 0.752, respectively. These nomograms showed superior performance compared with the FIGO 2018 staging criteria (*P* < 0.05).

**Conclusions:**

A novel prognostic nomogram score provides better prognostic performance than the FIGO 2018 staging system. These nomograms contribute to directing clinical treatment and prognosis assessment in patients harboring site-distant metastases.

**Supplementary Information:**

The online version contains supplementary material available at 10.1186/s12885-021-07977-4.

## Background

Epithelial ovarian cancer is a deadly malignant disease [1]. According to the SEER cancer statistics review (1975–2015), with more than twenty-thousand cases and fourteen-thousand deaths annually [2]. Although with the continuous advancement of surgical techniques and the improvement of chemotherapy drugs, the overall survival rate of patients with epithelial ovarian cancer has improved in the past 50 years, but the five-year overall survival rate is still less than 50% [3–5]. Numerous studies have investigated the association among potential survival trajectories as to prognosis of ovarian cancer [6]. However, the predictors of long-term survival are not well elucidated.

Sites-distant metastases seem to represent a significant cause of morbidity and mortality among patients with epithelial ovarian cancer [7–9]. Previous studies from case report and single institution experiences have yielded various conclusions, especially robust population-based estimation relating to the incidence of sites-distant metastases at diagnosis are common. However, populations-level estimation for prognosis among patients with newly diagnosed epithelial ovarian cancer and sites-distant metastases are also lacking.

In autopsy studies, patients with epithelial ovarian cancer are inclined to liver, lung, bone and/or brain metastases, not all of which are clinically apparent prior to death [9–11]. Thus, in patients with both localized and/or organ specific distant metastases, the risk of prognostic factors should be reassessment. Furthermore, patients diagnosed to have localized and/or distant metastases are suitable candidates for studies on postoperative adjuvant treatment, also which can help surgeons choose appropriate surgical measures for patients in advance.

Based on a lack of proven benefit, the purpose of this study was to use the SEER database to summarize the incidence proportion of organ specific distant metastases. Based on a nomogram score, we also sought to predict survival time, in which depend on independent risk factors that contribute to prognosis.

## Methods

### Population-based study inclusion and exclusion criteria

The SEER database includes information on epithelial ovarian cancer incidence, outcome, and treatment for approximately 30 % of the US population [12]. The SEER registries collect data on patient demographics, primary tumor site, tumor morphology, stage at diagnosis, and first course of treatment, and they follow up with patients for vital status. Within the SEER 18 registries in the November 2016 data submission, we identified 148,597 patients diagnosed as ovarian cancer from 1975 to 2016. Inclusion criteria: (1) Epithelial ovarian cancer confirmed by pathologic diagnosis, meeting the criteria of International Classification of Diseases for Oncology, 3rd ed. [8]. The primary site was C56.9 ovary, (2) Patients who had intact clinical information and survival outcomes, (3) Evidence of distant metastases at newly diagnosis, based on the commonly used International Federation of Gynecology and Obstetrics (FIGO) staging system, distant metastases included FIGO III-A, III-B, III-C, III-NOS and IV. Likewise, due to that the database included distant metastases variables, which contributed to the presence of metastatic position like bone, liver, brain and lung at time of diagnosis. Exclusion criteria: (1) Patients who were diagnosed at autopsy or via death certificate, (2) Patients with carcinoma for whom the presence or absence of distant metastases at diagnosis were unknown, (3) Patients who had unknown follow-up records. To examine the generalizability of the nomogram, a single institutional registry consisting of 3112 patients who received treatment between January 2012 and December 2019 from the department of Tongji Hospital in the People’s Republic of China was established. Ethical approval was obtained from participating institutions through the chairperson of the Tongji Hospital institutional review boards who waived the need for patient consent for this study when individual patient consent were not identified.

### Follow-up information and classification

Patients were stratified by sites of distant metastases, as well as other variables included age, race/ethnicity, marital status, etc. The clinicopathologic variables in the study are shown in Table s1.

### Incidence and survival analysis

The incidence proportions were calculated for patients with organ specific metastases identified at diagnosis. The primary endpoint was overall survival, defined as the time from newly diagnosis to death or loss of follow-up. Survival time was stratified as time (t) less than or equal to or more than 60 months (cut-off point,5-year). In addition to all-cause mortality, a separate analysis as to ovary cancer with distant metastases survival time including OS and CSS were conducted among women diagnosed after 2010, used to assess the risk of distant metastases related to survival outcomes.

### Statistical analysis

Multivariate Cox regression was performed to find covariates associated with increased all-cause mortality using the same variables as in the logistic regression model described herein. The stepwise regression based on the Akaike information criterion minimum was used to select variables for inclusion in the nomogram. Decision curve analysis (DCA) was used to determine whether the models could be considered useful tools for clinical decision making by comparing the net benefits at any threshold probability [13]. The 3- and 5-year OS and CSS probabilities were estimated using the nomogram. Concordance index (C-index) and area under the time-dependent receiver operating characteristic curve (time-dependent ROC) calculated by bootstrapping plots were used to compare the prognostic accuracy of the nomograms and the FIGO (2018) staging system. All analyses were performed using R software, version 3.6.2 (https://www.r-project.org/). *P* < 0.05 was considered statistical significance.

## Results

A total of 131,050 women with epithelial ovarian cancer were included in this study (Figure s1). 18.2, 7.8 and 66.1% had localized, regional and distant metastases, respectively. Among the cohort with metastatic disease, 18.6, 81.4% were aged≤50 and > 50 years, respectively. 30.0, 70.0% were survived > 60 and ≤ 60 months, respectively. Compared to less than 5-year overall survival patients, most survivors with long-term (> 60 months) were more likely to be diagnosed with localized metastases, as well as younger age, lower grade and accepting surgery (Table s1).

### Incidence and median survival of patients with metastases

Among the patients in the all-cause mortality cohort(*n* = 33,727) and cancer-specific mortality cohort (*n* = 15,742) with metastases at diagnosis were identified (Table s2). For incidence, 0.9, 6.7, 5.8 and 0.2% had bone, liver, lung and brain metastases in the all-cause mortality cohort, respectively, 1.6, 9.9, 8.8 and 0.3% had bone, liver, lung and brain metastases in the cancer-specific mortality cohort, respectively. The incidence proportion of patients with organ-specific or solitary metastases at diagnosis provided in Table 1. As stratified by organ-site metastases, the median survival among the all-cause mortality cohort was 21.0 months, cancer-specific mortality cohort was 15.0 months. As stratified by organ-specific metastases and solitary organ metastases, patients with solitary lung metastases showed the longest median survival (11.0 months) and patients with brain metastases had the shortest median survival (3.5 months) in the all-cause mortality cohort. Patients with solitary lung metastases showed the longest median survival (8.0 months) and patients with solitary lymph metastases experienced the shortest median survival (1.5 months).

**Table 1:** Incidence proportion and median survival in patients with metastases at diagnosis

Metastases subtype	Incidence Proportion of all-cause mortality cohort, %	Median survival (IQR), month	Incidence Proportion of cancer-specific mortality cohort, %	Median survival (IQR), month
Bone	0.9	5.0 (1.0–11.0)	1.6	4.0 (1.0–9.0)
Liver	6.7	9.0 (2.0–25.0)	9.9	6.0 (1.0–19.0)
Lung	5.8	9.0 (2.0–24.0)	8.8	6.0 (1.0–21.0)
Brain	0.2	3.5 (1.0–11.0)	0.3	3.0 (1.0–7.0)
Lymph	0.9	4.0 (1.0–7.0)	0.5	1.5 (1.0–5.75)
Bone only	0.4	7.0 (2.0–19.5)	0.6	6.0 (2.0–14.5)
Liver only	0.1	10.0 (2.0–28.0)	6.9	7.0 (1.0–20.0)
Lung only	3.9	11.0 (2.0–25.0)	5.8	8.0 (1.0–22.0)
Lymph only	0.5	5.0 (2.0–8.0)	0.2	2.0 (1.0–4.5)
Brain only	4.8	4.0 (2.0–12.0)	0.2	2.5 (2.0–7.75)

*Abbreviations*: *IQR* Interquartile range

### Risk factors screening for predicting long-term survival possibility

After multivariable adjustment, the risk of 5-year overall survival was significantly associated with race (HR,1.09;95%CI,1.06–1.13), grade (III/IV: HR,1.03;95%CI,0.99–1.07), histology (HR,0.95;95%CI,0.92–0.98), FIGO (II: HR,0.97;95%CI,0.92–1.03;III: HR,0.86;95%CI,0.83–0.90; IV: HR,0.83;95%CI,0.78–0.88); bone metastasis (HR,0.77;95%CI,0.61–0.96); brain metastasis (HR,0.77;95%CI,0.47–1.26); liver metastasis (HR,0.87;95%CI,0.80–0.95); lung metastasis (HR,0.87;95%CI,0.80–0.96); lymphatic metastasis (HR,0.47;95%CI,0.41–0.54); surgery (HR,0.75;95%CI,0.71–0.80) among the all-cause mortality cohort (Table 2). Among the cancer specific mortality cohort, we observed similar, although not statistically significant, association for organ-specific metastasis. Lung metastasis (HR,0.80;95%CI,0.48–1.32), brain metastasis (HR,1.63;95%CI,0.20–13.30), bone metastasis (HR,0.88;95%CI,0.27–2.89), lymphatic metastasis (HR,0.47;95%CI,0.41–0.54) were associated with a significant decrease in long-term survival possibility (Table 3).

**Table 2:** Multivariate Cox regression analysis of survival in overall survival

Variables	OS			
	HR	95%CI lower	95%CI upper	*P* value
Age, y^a^	1.00	0.99	1.01	0.37
Race				
White	1[ref]	NA	NA	
Non-white	1.09	1.06	1.13	< 0.01
Grade				
I/II	1[ref]	NA	NA	
III/IV	1.03	0.99	1.07	< 0.01
Unknown	0.89	0.85	0.93	< 0.01
Histology				
Serous	1[ref]	NA	NA	
Non-serous	0.95	0.92	0.98	< 0.01
FIGO				
I	1[ref]	NA	NA	
II	0.97	0.92	1.03	< 0.01
III	0.86	0.83	0.90	< 0.01
IV	0.83	0.78	0.88	< 0.01
Unknown	10.46	9.98	10.96	< 0.01
Metastases				
Bone				
Yes	1[ref]	NA	NA	
No	0.77	0.61	0.96	< 0.01
Brain				
Yes	1[ref]	NA	NA	
No	0.77	0.47	1.26	0.03
Liver				
Yes	1[ref]	NA	NA	
No	0.87	0.80	0.95	< 0.01
Lung				
Yes	1[ref]	NA	NA	
No	0.87	0.80	0.96	0.02
Lymph				
Yes	1[ref]	NA	NA	
No	0.47	0.41	0.54	< 0.01
Insurance				
Yes	1[ref]	NA	NA	
No	1.05	1.00	1.08	0.35
Surgery				
Yes	1[ref]	NA	NA	
No	0.75	0.71	0.80	< 0.01
Martial				
Yes	1[ref]	NA	NA	
No	1.02	0.99	1.06	0.17

^a^Continuous variable

**Table 3:** Multivariate Cox regression analysis of survival in cancer specific survival

Variables	CSS			
	HR	95%CI lower	95%CI upper	*P* value
Race				
White	1[ref]	NA	NA	
Non-white	0.91	0.77	1.09	< 0.01
Grade				
I/II	1[ref]	NA	NA	
III/IV	1.30	1.06	1.60	< 0.01
Unknown	0.95	0.77	1.17	< 0.01
Site				
Localized	1[ref]	NA	NA	
Distant	0.78	0.66	0.94	0.04
FIGO				
I	1[ref]	NA	NA	
II	1.58	1.26	1.97	< 0.01
III	1.26	0.97	1.63	< 0.01
IV	0.75	0.58	0.97	< 0.01
Unknown	0.10	0.06	0.17	< 0.01
Metastases				
Bone				
Yes	1[ref]	NA	NA	
No	0.88	0.27	2.89	< 0.01
Brain				
Yes	1[ref]	NA	NA	
No	1.63	0.20	13.30	0.03
Liver				
Yes	1[ref]	NA	NA	
No	1.06	0.69	1.63	0.80
Lung				
Yes	1[ref]	NA	NA	
No	0.80	0.48	1.32	< 0.01
Lymph				
Yes	1[ref]	NA	NA	
No	0.47	0.41	0.54	< 0.01
Insurance				
Yes	1[ref]	NA	NA	
No	0.15	0.12	0.17	< 0.01
Surgery				
Yes	1[ref]	NA	NA	
No	0.18	0.11	0.27	< 0.01
Martial				
Yes	1[ref]	NA	NA	
No	0.84	0.68	1.04	0.17

### Development and validation of a prognosis predicting nomogram

**Fig. 1:**
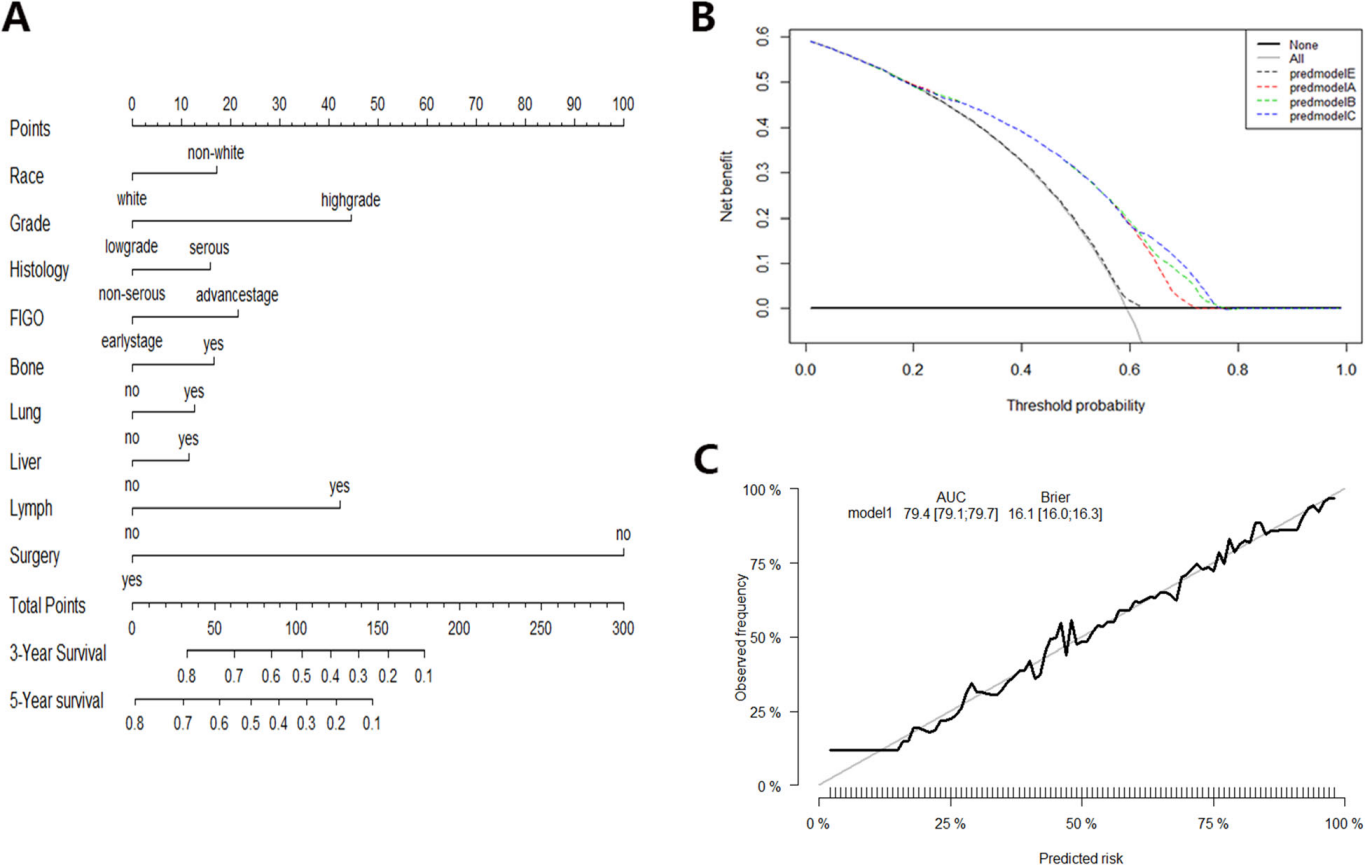
Nomogram score for OS probability estimation. **a** Nomogram to estimate the risk of 5-year OS in patients with sites-distant metastases. **b** Decision curve for the prediction of 5-year OS. Decision curve analysis identified potential factors that can exert clinical influence based on stepwise regression analysis and the net benefit of using nomogram score to stratify patients. Four models were built before the final nomogram was constructed: predmodelA (FIGO, Grade, Race, and Age), predmodelB (FIGO, Grade, Race, Surgery, and site-distant metastases), predmodelC (FIGO, age), and predmodelE (FIGO, Grade, Race, and Histology). **c** The calibration curve for predicting 5-year OS survival. Nomogram-predicted risk of overall survival is showed on the x-axis, actual overall survival is mirrored on the y-axis

**Fig. 2:**
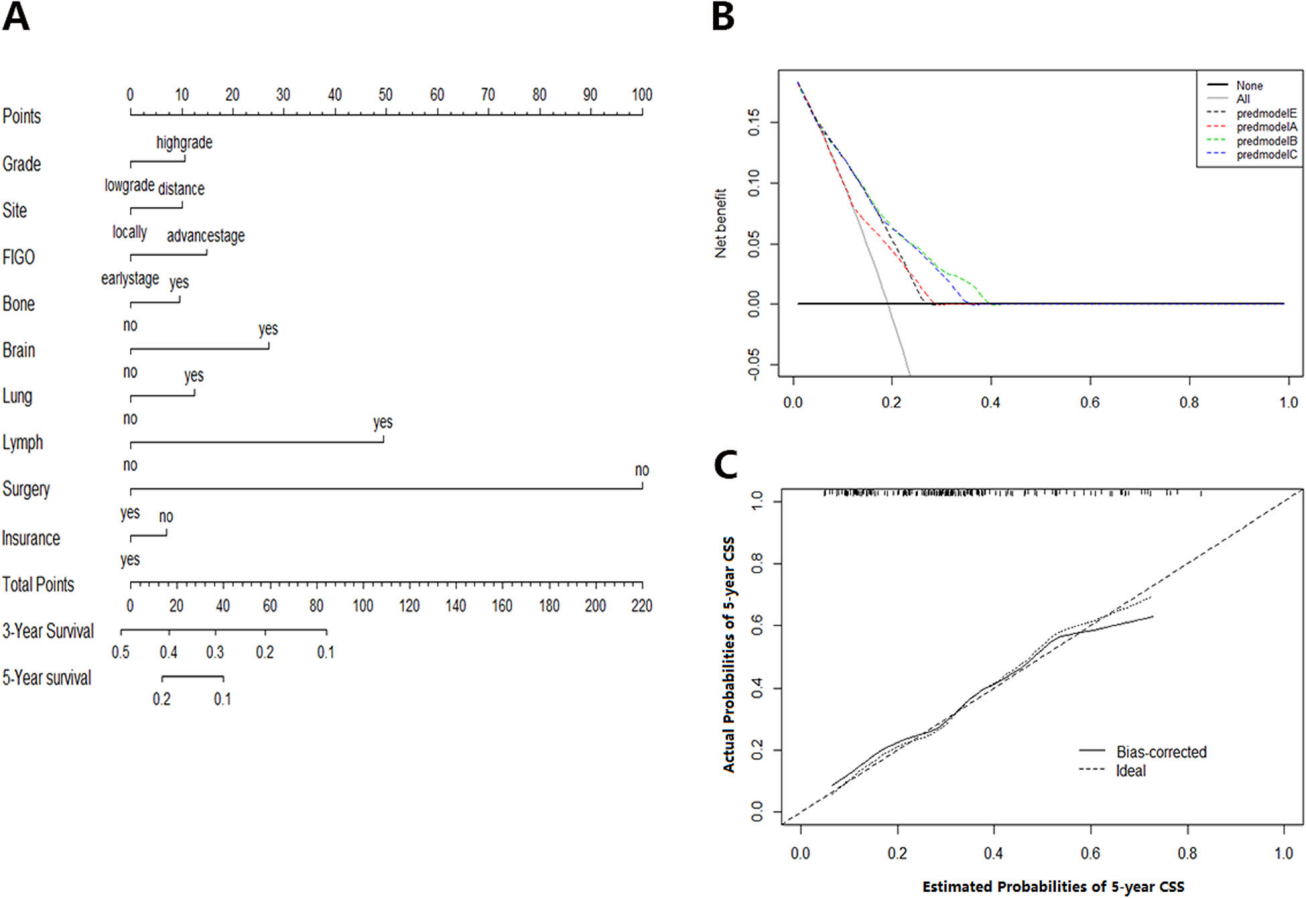
Nomogram score for CSS probability estimation. **a** Nomogram to estimate the risk of CSS in patients with sites-distant metastases. **b** Decision curve for the prediction of CSS. Decision curve analysis identified potential factors that can exert clinical influence based on stepwise regression analysis and the net benefit of using nomogram score to stratify patients. Four models were built before the final nomogram was constructed: predmodelA (FIGO, Grade, Site, and Surgery), predmodelB (FIGO, Grade, Site, and Surgery, and site-distant metastases), predmodelC (FIGO, Grade, and Surgery), and predmodelE (FIGO, Grade, Site, Surgery, and Insurance). **c** The calibration curve for predicting 5-year CSS survival. Nomogram-predicted risk of cancer specific survival is showed on the x-axis, actual cancer specific survival is mirrored on the y-axis

**Fig. 3:**
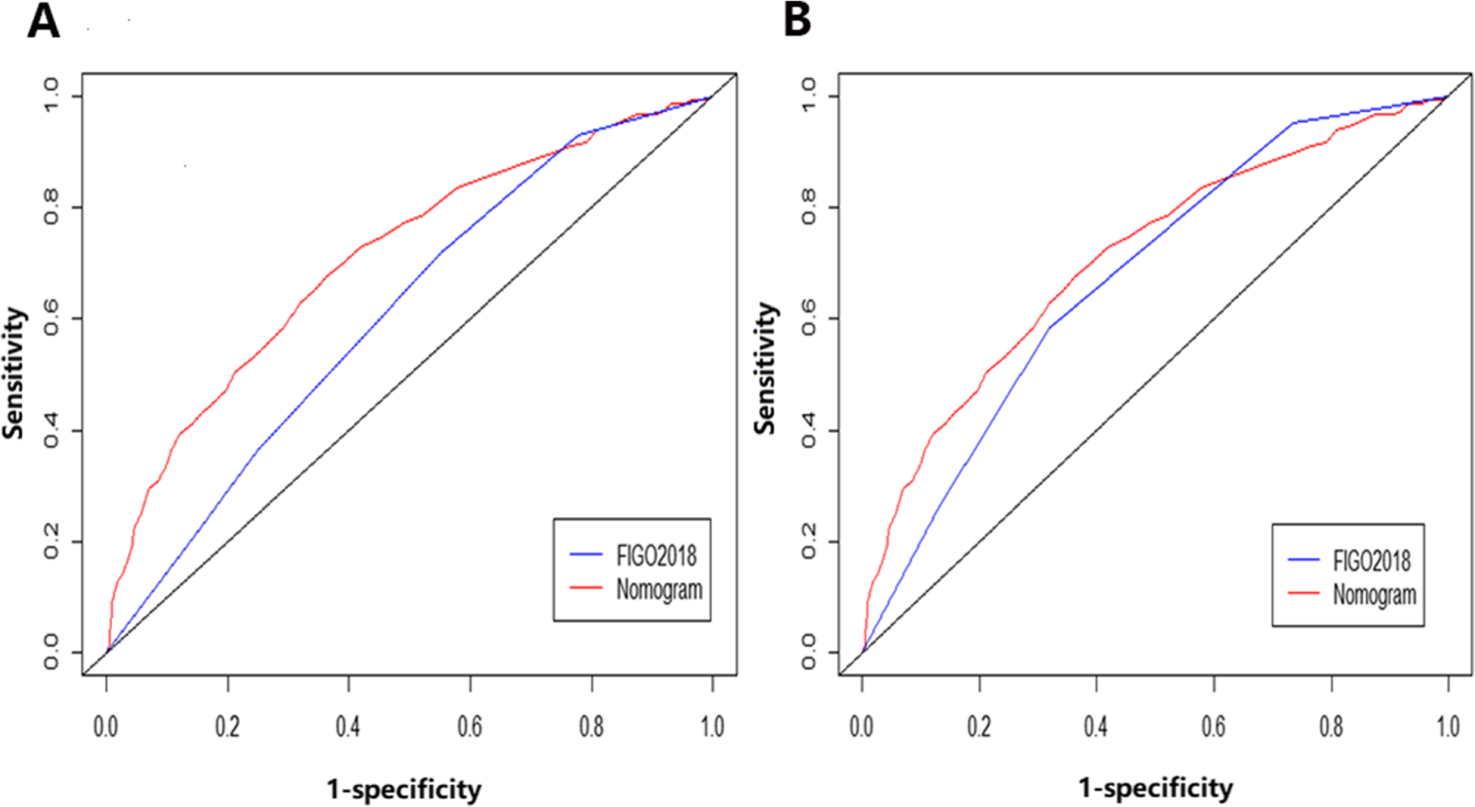
Comparison of nomogram score and FIGO 2018 staging system. **a** The AUC for predicting 5-year CSS. **b** The AUC for predicting 5-year OS

On multivariate Cox regression for all-cause mortality and cancer-specific mortality, organ-specific metastases were independently associated with survival prognosis (Table 2). These independent associated risk factors were performed to form a prognosis (3-year/5-year overall survival) estimation nomogram. Among the all-cause mortality cohort, 70% of patients were used for training, 30% of patients were used for validation. So did the cancer-specific mortality cohort. The nomogram score demonstrated good accuracy in estimating the prognosis of 3-year and 5-year OS (Fig. 1). In the all-cause mortality training and validation cohort, a bootstrap-corrected C index was 0.791,0.788, respectively. The calibration plot also showed robust result of the estimation (Fig. 1). In the cancer-specific mortality training and validation cohort, the nomogram displayed C index was 0.752, 0.745, respectively (Fig. 2). Additionally, the results from external datasets are consistent with those from internal datasets. The nomogram for OS had a significantly higher AUC compared with the FIGO 2018 staging system (0.709 vs 0.676. *P* < 0.05). Similarly, the AUC of the nomogram for predicting CSS was significantly superior compared with the FIGO 2018 staging system (0.708 vs 0.610. *P* < 0.05) (Fig. 3).

## Discussion

Epithelial ovarian cancer is the most common pathological type in ovarian malignant tumors [5]. Based on the SEER cancer registry data analysis, the potential factors of long-term survival were comprehensively understood in this study. Taken the 5-year overall survival as a cut-off point, we observed that site of metastases and organ-specific metastases strongly were associated with overall survival in the all-cause mortality cohort and cancer-specific mortality cohort, these survival trajectories potentially contributed to patients prognosis with distant metastases, especially among patients with organ-specific metastases at diagnosis. Likewise, consistent with previous studies, other survival trajectories such as race, ovarian involvement, histology, age (taken menopause age as borderline), insurance and marital status, which were commonly relevant to the long-term prognosis [14–16]. In addition, this study explored the risk of 5-year overall survival among patients with localized, regional and distant metastases, our data demonstrated that distant metastases were significantly associated with long-term survival (> 5 years). Collectively, tumor migration may contribute to the worse long-term survival in patients with site-distant metastases at initial diagnosis. To the best of our knowledge, this is the largest population-based study exploring prognosis in patients with epithelial ovarian cancer diagnosed with tumor metastases at diagnosis.

Our study reported the incidence and median survival time of different organ-specific metastases. Consistent with some but not all prior studies [8, 11, 17], liver metastasis showed the highest incidence in all-cause mortality cohorts, followed by lung metastasis, bone metastasis and brain metastasis had relatively low incidence.

However, in solitary organ metastasis, the incidence of brain and lung metastases were relatively high. In the cancer-specific death cohort, we found that the same rules followed, except that the incidence of solitary lung metastasis and liver metastasis were higher than the former cohort. Interestingly, despite the high incidence of lung and liver metastases, the median survival time for these patients was relatively longer. Up to now, there is no standard treatment plan for distant metastasis. The main treatment is to control the primary disease. The choice of treatment plan needs to fully evaluate the patient’s condition [18, 19]. In general, although the incidence of organ-specific metastasis is not high in the entire cohort, which have potential impact on the prognosis of patients, so attention should be paid to the decision of the treatment plan for patients with distant metastases at the initial diagnosis.

In previous studies, the prognostic assessment of patients with epithelial ovarian cancer usually used postoperative FIGO staging, pathological tissue typing, and lymphatic metastasis as evaluation criteria [20–22]. Zou et al. reported that UBE2T could serve as a new prognostic marker and therapeutic target for this disease [23]. However, with the optimization and improvement of treatment methods, more and more patients already have site-distant metastases at initial diagnosis, so it is necessary to adequately evaluate the prognosis of patients by combining the risk of different site-distant metastases. Our study found that organ metastasis had a significant impact on the 5-year overall survival, especially lung, liver and bone metastases, consistent with previous reports [18, 24, 25].

Compared with lymphatic metastasis, patients with organ metastases have a relatively lower 5-year overall survival and worse prognosis. Therefore, our study adopted the nomogram scoring standard to quantify the metastasis of different organs, and assign points based on the specific location of the metastasis at the initial diagnosis of the patient, so as to effectively evaluate the 3-year overall survival time and 5-year overall survival time. In the process of establishing the nomogram, we used regression analysis to screen out 14 variables significantly related to prognosis. The model was robust through 1000 consecutive iteration tests. At the same time, the calibration curve also showed that the predicted value and the actual consistency.

The use of the nomogram score in estimating the risk of a patient harboring organ-specific metastases to direct clinical treatment and prognosis assessment is a novel concept. Because there are many factors affecting the prognosis of patients, the risk of metastases is worth considering. Other factors such as age, race, surgery, pathological type, FIGO staging system, insurance, and marital status also should be considered. In short, we have established a visual prognostic evaluation model, as well as beneficial reference value for guiding patients’ treatment and prognosis.

Our research also has the following limitations. First, we use the SEER database to assess the prognostic risk of patients. To the best of our knowledge, tumor metastases that affect the prognosis of patients should also consider many factors such as retroperitoneal lymph nodes and chemotherapy. This information cannot be obtained from the database. Second, there are some patients who may be at risk of distant metastases during treatment. We cannot accurately obtain detailed information about these patients, so the risk of recurrence cannot be assessed. Third, laboratory indicators such as CA125 are related to the prognosis of ovarian cancer patients, but the database has only partial records, so it was not included in this study. Finally, we cannot obtain a detailed treatment plan for the patient, so we cannot assess the potential influencing factors of the prognosis assessment.

## Conclusion

Despite the above limitations, our study depended on the visual scale of organ metastases scale for the first time to quantitatively evaluate the 5-year survival risk of patients, for which accurately predict the 3-year survival time and 5-year survival time of patients with different organ metastases. For patients with initial diagnosis of organ metastasis, the prognostic evaluation criteria should focus on organ-specific metastases at diagnosis, which is worthy of further research and confirmation.

## Supplementary Information

**Additional file 1: Figure s1.** Flow diagram with selection procedure of patients with epithelial ovarian cancer for analysis.

**Additional file 2: Table s1.** Demographics and Clinicopathologic Characteristics of Patients with Epithelial Ovarian Cancer.

**Additional file 3: Table s2.** Demographics and Clinicopathologic Characteristics of Patients with Epithelial Ovarian Cancer in the OS and CSS cohort. Abbreviations: OS, overall survival. CSS, cancer specific survival.

## Data Availability

The datasets used and/or analysed during the current study are available from the corresponding author on reasonable request.

## Abbreviations

SEER: Surveillance, Epidemiology, and End Results Program
OS: Overall survival (equivalent to, all-cause mortality)
CI: Confidence interval
HR: Hazard ratio
C-index: Concordance index
AUC: Area under the curve
tdROC: Time dependent receiver operator characteristic curve
FIGO: International Federation of Gynecology and Obstetrics
CSS: Cancer-specific survival (equivalent to, cancer-specific mortality)
