# Supplementary material for: Development and validation of a nomogram to predict survival outcome among epithelial ovarian cancer patients with site-distant metastases: a population-based study
Source: BMC Cancer. 2021 May 25;21:609. doi: 10.1186/s12885-021-07977-4 (PMC8152065; doi:10.1186/s12885-021-07977-4)
Supplement: Supplementary file 1 — Additional file 1: Figure s1. Flow diagram with selection procedure of patients with epithelial ovarian cancer for analysis. [file 12885_2021_7977_MOESM1_ESM.docx]

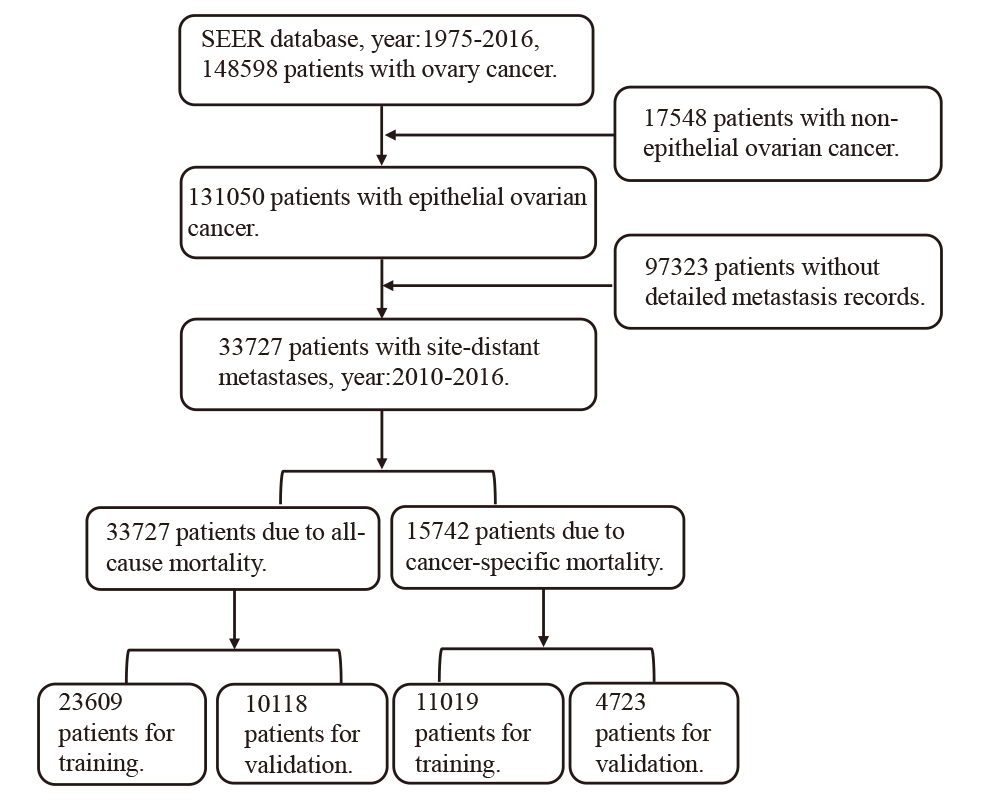


Figures1. Flow diagram with selection procedure of patients with epithelial ovarian cancer for analysis
