# Supplementary material for: Development and validation of a nomogram to predict survival outcome among epithelial ovarian cancer patients with site-distant metastases: a population-based study
Source: BMC Cancer. 2021 May 25;21:609. doi: 10.1186/s12885-021-07977-4 (PMC8152065; doi:10.1186/s12885-021-07977-4)
Supplement: Supplementary file 2 — Additional file 2: Table s1. Demographics and Clinicopathologic Characteristics of Patients with Epithelial Ovarian Cancer. [file 12885_2021_7977_MOESM2_ESM.docx]

Table s1. Demographics and Clinicopathologic Characteristics of Patients with Epithelial Ovarian Cancer

| Variable | Age≤50y | | | |  | Age＞50y | | | |
| --- | --- | --- | --- | --- | --- | --- | --- | --- | --- |
|  | localized | regional | distant | unknown |  | localized | regional | distant | unknown |
|  | (n=15657) | (n=8086) | (n=74106) | (n=8863) |  | (n=8158) | (n=2080) | (n=12575) | (n=1525) |
| Age, y |  |  |  |  |  |  |  |  |  |
| ≤50 | 15657 (100%) | 8086 (100%) | 74106 (100%) | 8863 (100%) |  | 0 (0%) | 0 (0%) | 0 (0%) | 0 (0%) |
| >50 | 0 (0%) | 0 (0%) | 0 (0%) | 0 (0%) |  | 8158 (100%) | 2080 (100%) | 12575 (100%) | 1525 (100%) |
| Age1, y |  |  |  |  |  |  |  |  |  |
| mean(±SD) | 63.7(±10.2) | 66.2(±10.9) | 68.1(±10.6) | 71.0(±11.9) |  | 39.9(±7.67) | 42.4(±6.40) | 42.4(±6.36) | 40.6(±7.58) |
| min, max | 50.0, 101 | 50.0, 100 | 50.0, 113 | 50.0, 102 |  | 9.00, 49.0 | 12.0, 49.0 | 12.0, 49.0 | 12.0, 49.0 |
| median (Q1, Q3) | 62.0(55.0, 71.0) | 65.0(57.0, 74.0) | 68.0(60.0, 76.0) | 70.0(61.0, 81.0) |  | 42.0(35.0, 46.0) | 44.0(40.0, 47.0) | 44.0(40.0, 47.0) | 43.0(37.0, 47.0) |
| Survival, month |  |  |  |  |  |  |  |  |  |
| mean(±SD) | 113(±93.6) | 70.6(±73.7) | 37.0(±51.5) | 18.7(±42.5) |  | 163(±126) | 109(±99.3) | 74.0(±91.5) | 53.2(±89.8) |
| min, max | 0.00, 495 | 0.00, 493 | 0.00, 503 | 0.00, 479 |  | 0.00, 503 | 0.00, 500 | 0.00, 503 | 0.00, 498 |
| median (Q1, Q3) | 90.0(40.0, 162) | 46.0(18.0, 100) | 20.0(5.00, 45.0) | 5.00(1.00, 11.0) |  | 136(60.0, 240) | 77.5(34.0, 154) | 40.0(17.0, 91.0) | 9.00(4.00, 61.0) |
| Race |  |  |  |  |  |  |  |  |  |
| Hispanic (All Races) | 1175 (7.5%) | 649 (8.0%) | 5667 (7.6%) | 961 (10.8%) |  | 1028 (12.6%) | 300 (14.4%) | 1826 (14.5%) | 295 (19.3%) |
| Non-Hispanic American Indian/Alaska Native | 74 (0.5%) | 31 (0.4%) | 403 (0.5%) | 44 (0.5%) |  | 55 (0.7%) | 10 (0.5%) | 112 (0.9%) | 9 (0.6%) |
| Non-Hispanic Asian or Pacific Islander | 1179 (7.5%) | 515 (6.4%) | 3726 (5.0%) | 560 (6.3%) |  | 992 (12.2%) | 255 (12.3%) | 1177 (9.4%) | 171 (11.2%) |
| Non-Hispanic Black | 769 (4.9%) | 522 (6.5%) | 5189 (7.0%) | 778 (8.8%) |  | 407 (5.0%) | 159 (7.6%) | 1044 (8.3%) | 156 (10.2%) |
| Non-Hispanic Unknown Race | 41 (0.3%) | 19 (0.2%) | 52 (0.1%) | 41 (0.5%) |  | 45 (0.6%) | 9 (0.4%) | 32 (0.3%) | 21 (1.4%) |
| Non-Hispanic White | 12419 (79.3%) | 6350 (78.5%) | 59069 (79.7%) | 6479 (73.1%) |  | 5631 (69.0%) | 1347 (64.8%) | 8384 (66.7%) | 873 (57.2%) |
| Grade |  |  |  |  |  |  |  |  |  |
| I | 3284 (21.0%) | 519 (6.4%) | 1885 (2.5%) | 294 (3.3%) |  | 2419 (29.7%) | 273 (13.1%) | 966 (7.7%) | 195 (12.8%) |
| II | 3907 (25.0%) | 1238 (15.3%) | 7649 (10.3%) | 452 (5.1%) |  | 2009 (24.6%) | 475 (22.8%) | 2097 (16.7%) | 198 (13.0%) |
| III | 3065 (19.6%) | 3011 (37.2%) | 26675 (36.0%) | 1402 (15.8%) |  | 996 (12.2%) | 622 (29.9%) | 4615 (36.7%) | 219 (14.4%) |
| IV | 1079 (6.9%) | 1234 (15.3%) | 9663 (13.0%) | 1151 (13.0%) |  | 366 (4.5%) | 238 (11.4%) | 1628 (12.9%) | 172 (11.3%) |
| unknown | 4322 (27.6%) | 2084 (25.8%) | 28234 (38.1%) | 5564 (62.8%) |  | 2368 (29.0%) | 472 (22.7%) | 3269 (26.0%) | 741 (48.6%) |
| Site |  |  |  |  |  |  |  |  |  |
| unilateral | 13994 (89.4%) | 5411 (66.9%) | 28396 (38.3%) | 3445 (38.9%) |  | 7357 (90.2%) | 1349 (64.9%) | 4439 (35.3%) | 821 (53.8%) |
| bilateral | 1663 (10.6%) | 2675 (33.1%) | 45710 (61.7%) | 5418 (61.1%) |  | 801 (9.8%) | 731 (35.1%) | 8136 (64.7%) | 704 (46.2%) |
| Histology |  |  |  |  |  |  |  |  |  |
| serous | 4178 (26.7%) | 3827 (47.3%) | 42422 (57.2%) | 3153 (35.6%) |  | 1652 (20.3%) | 799 (38.4%) | 7559 (60.1%) | 519 (34.0%) |
| endometrioid | 4200 (26.8%) | 1345 (16.6%) | 3784 (5.1%) | 454 (5.1%) |  | 2442 (29.9%) | 608 (29.2%) | 1309 (10.4%) | 233 (15.3%) |
| mucinous | 2972 (19.0%) | 425 (5.3%) | 3014 (4.1%) | 381 (4.3%) |  | 2254 (27.6%) | 153 (7.4%) | 841 (6.7%) | 175 (11.5%) |
| clear cell | 2075 (13.3%) | 531 (6.6%) | 1789 (2.4%) | 290 (3.3%) |  | 921 (11.3%) | 186 (8.9%) | 597 (4.7%) | 109 (7.1%) |
| carcinosarcoma | 274 (1.8%) | 395 (4.9%) | 2487 (3.4%) | 255 (2.9%) |  | 85 (1.0%) | 40 (1.9%) | 201 (1.6%) | 24 (1.6%) |
| Brenner | 1293 (8.3%) | 1196 (14.8%) | 19104 (25.8%) | 4149 (46.8%) |  | 484 (5.9%) | 176 (8.5%) | 1665 (13.2%) | 419 (27.5%) |
| mixed | 665 (4.2%) | 367 (4.5%) | 1506 (2.0%) | 181 (2.0%) |  | 320 (3.9%) | 118 (5.7%) | 403 (3.2%) | 46 (3.0%) |
| Metastases |  |  |  |  |  |  |  |  |  |
| localized | 15657 (100%) | 0 (0%) | 0 (0%) | 0 (0%) |  | 8158 (100%) | 0 (0%) | 0 (0%) | 0 (0%) |
| regional | 0 (0%) | 8086 (100%) | 0 (0%) | 0 (0%) |  | 0 (0%) | 2080 (100%) | 0 (0%) | 0 (0%) |
| distant | 0 (0%) | 0 (0%) | 74106 (100%) | 0 (0%) |  | 0 (0%) | 0 (0%) | 12575 (100%) | 0 (0%) |
| unknown | 0 (0%) | 0 (0%) | 0 (0%) | 8863 (100%) |  | 0 (0%) | 0 (0%) | 0 (0%) | 1525 (100%) |
| Surgery |  |  |  |  |  |  |  |  |  |
| yes | 15426 (98.5%) | 7249 (89.6%) | 53446 (72.1%) | 4284 (48.3%) |  | 8109 (99.4%) | 2020 (97.1%) | 11502 (91.5%) | 1180 (77.4%) |
| no | 231 (1.5%) | 837 (10.4%) | 20660 (27.9%) | 4579 (51.7%) |  | 49 (0.6%) | 60 (2.9%) | 1073 (8.5%) | 345 (22.6%) |
| Survival time, month |  |  |  |  |  |  |  |  |  |
| ≤60 | 5688 (36.3%) | 4787 (59.2%) | 60961 (82.3%) | 8155 (92.0%) |  | 2065 (25.3%) | 863 (41.5%) | 8077 (64.2%) | 1141 (74.8%) |
| >60 | 9969 (63.7%) | 3299 (40.8%) | 13145 (17.7%) | 708 (8.0%) |  | 6093 (74.7%) | 1217 (58.5%) | 4498 (35.8%) | 384 (25.2%) |
| Status |  |  |  |  |  |  |  |  |  |
| dead | 7241 (46.2%) | 4803 (59.4%) | 62575 (84.4%) | 5000 (56.4%) |  | 1520 (18.6%) | 739 (35.5%) | 8521 (67.8%) | 409 (26.8%) |
| alive | 8416 (53.8%) | 3283 (40.6%) | 11531 (15.6%) | 3863 (43.6%) |  | 6638 (81.4%) | 1341 (64.5%) | 4054 (32.2%) | 1116 (73.2%) |
| Marital |  |  |  |  |  |  |  |  |  |
| married | 12802 (81.8%) | 6695 (82.8%) | 63226 (85.3%) | 6998 (79.0%) |  | 5375 (65.9%) | 1414 (68.0%) | 8951 (71.2%) | 928 (60.9%) |
| non-married | 2288 (14.6%) | 1064 (13.2%) | 8503 (11.5%) | 1302 (14.7%) |  | 2490 (30.5%) | 606 (29.1%) | 3304 (26.3%) | 476 (31.2%) |
| unknown | 567 (3.6%) | 327 (4.0%) | 2377 (3.2%) | 563 (6.4%) |  | 293 (3.6%) | 60 (2.9%) | 320 (2.5%) | 121 (7.9%) |
