# Supplementary material for: Development and validation of a nomogram to predict survival outcome among epithelial ovarian cancer patients with site-distant metastases: a population-based study
Source: BMC Cancer. 2021 May 25;21:609. doi: 10.1186/s12885-021-07977-4 (PMC8152065; doi:10.1186/s12885-021-07977-4)
Supplement: Supplementary file 3 — Additional file 3: Table s2. Demographics and Clinicopathologic Characteristics of Patients with Epithelial Ovarian Cancer in the OS and CSS cohort. Abbreviations: OS, overall survival. CSS, cancer specific survival. [file 12885_2021_7977_MOESM3_ESM.docx]

Tables2. Demographics and Clinicopathologic Characteristics of Patients with Epithelial Ovarian Cancer in the OS and CSS cohort

| Variables | OS | CSS |
| --- | --- | --- |
|  | (n=33727) | (n=15742) |
| Age, y |  |  |
| ＜50 | 5898 (17.5%) | 1719 (10.9%) |
| ≥50 | 27829 (82.5%) | 14023 (89.1%) |
| Race |  |  |
| White | 23447 (69.5%) | 11278 (71.6%) |
| Non-white | 10280 (30.5%) | 4464 (28.4%) |
| Grade |  |  |
| I-II | 6089 (18.1%) | 1720 (10.9%) |
| III-IV | 16739 (49.6%) | 7522 (47.8%) |
| Unknown | 10899 (32.3%) | 6500 (41.3%) |
| Site |  |  |
| Unilateral | 17700 (52.5%) | 6807 (43.2%) |
| Bilateral | 16027 (47.5%) | 8935 (56.8%) |
| Metastases |  |  |
| Bone |  |  |
| Yes | 318 (0.9%) | 247 (1.6%) |
| No | 33409 (99.1%) | 15495 (98.4%) |
| Brain |  |  |
| Yes | 74 (0.2%) | 55 (0.3%) |
| No | 33653 (99.8%) | 15687 (99.7%) |
| Liver |  |  |
| Yes | 2249 (6.7%) | 1554 (9.9%) |
| No | 31478 (93.3%) | 14188 (90.1%) |
| Lung |  |  |
| Yes | 1952 (5.8%) | 1387 (8.8%) |
| No | 31775 (94.2%) | 14355 (91.2%) |
| Lymph |  |  |
| Yes | 299 (0.9%) | 86 (0.5%) |
| No | 33428 (99.1%) | 15656 (99.5%) |
| Solitary metastases |  |  |
| Bone |  |  |
| Yes | 134 (0.4%) | 96 (0.6%) |
| No | 33593 (99.6%) | 15646 (99.4%) |
| Brain |  |  |
| Yes | 37 (0.1%) | 24 (0.2%) |
| No | 33690 (99.9%) | 15718 (99.8%) |
| Liver |  |  |
| Yes | 1633 (4.8%) | 1086 (6.9%) |
| No | 32094 (95.2%) | 14656 (93.1%) |
| Lung |  |  |
| Yes | 1325 (3.9%) | 911 (5.8%) |
| No | 32402 (96.1%) | 14831 (94.2%) |
| survival |  |  |
| Mean(±SD) | 26.7(±22.8) | 20.5(±19.8) |
| Min, Max | 0.00, 83.0 | 0.00, 83.0 |
| Median (Q1, Q3) | 21.0(7.00, 42.0) | 15.0(4.00, 32.0) |
| status |  |  |
| Yes | 20045 (59.4%) | 3011 (19.1%) |
| No | 13682 (40.6%) | 12731 (80.9%) |

Abbreviations: OS, overall survival. CSS, cancer specific survival.
